# Eustachian Valve Endocarditis: Echocardiographic Diagnosis in a Critical Care Patient

**DOI:** 10.1155/2018/5193976

**Published:** 2018-01-31

**Authors:** Mariana Alves, Rita Faria, António Messias, Carlos Meneses-Oliveira

**Affiliations:** ^1^Hospital Pulido Valente, Medicina III, Lisboa, Portugal; ^2^Faculdade de Medicina da Universidade de Lisboa, Lisboa, Portugal; ^3^Hospital Beatriz Ângelo, Unidade de Cuidados Intensivos, Loures, Portugal

## Abstract

Eustachian valve endocarditis is rare. A literature review revealed that only 29 cases have been reported and, among them, there is only one mention of an intensive care unit (ICU) admission. We present an 82-year-old man without previous medical records who presented with septic shock with multiple organ dysfunction. The patient was admitted to the ICU and deteriorated with combined shock (septic + cardiogenic). A second ultrasound screen detected a prominent Eustachian valve with mobile multilobulated vegetation attached. Transesophageal echocardiography confirmed a 12 mm oscillating mass attached to a visible Eustachian valve.

## 1. Introduction

Eustachian valve endocarditis (EVE) was firstly described in 1986. From 1986 till 2015, only 29 cases have been reported, and there is only one mention of ICU admission [1].

A recent literature review showed that the main predisposing factor is intravenous drug use and the causative agent is* Staphylococcus aureus*. Other common causes are indwelling catheters, rheumatic heart disease, pacemaker wires, and immunologic compromise [1].

We report the case of an 82-year-old patient without intravenous drug use, admitted for septic shock with multiple organ dysfunction.

## 2. Case Report

An 82-year-old man without previous history of human immunodeficiency viral infection or intravenous drug use was admitted to the hospital with dyspnea and dry cough. He had been complaining of increased fatigability for several weeks. On admission, physical examination presented signs of hypoperfusion, fever, hypoxemia, and bilateral leg edema. Blood tests showed an elevated white blood cell count and C-reactive protein, acute kidney injury, and hepatic dysfunction with prolonged activated partial thromboplastin time and prothrombin time. Computed tomography pulmonary angiogram excluded central pulmonary embolism. A septic shock with multiple organ dysfunction was assumed. He was given empirical antibiotic treatment.

Transthoracic echocardiography (TTE) detected severe aortic stenosis with reduced ejection fraction, mild mitral regurgitation, and mild pulmonary hypertension. It was neglectful of suspicious images of endocarditis.

The patient was admitted in the ICU and required mechanical ventilation, inotropic support, and sustained low-efficiency dialysis. After orotracheal intubation, patient presented cardiorespiratory arrest that recovered spontaneous circulation after 6 minutes of advance life support.

**Figure 1:**
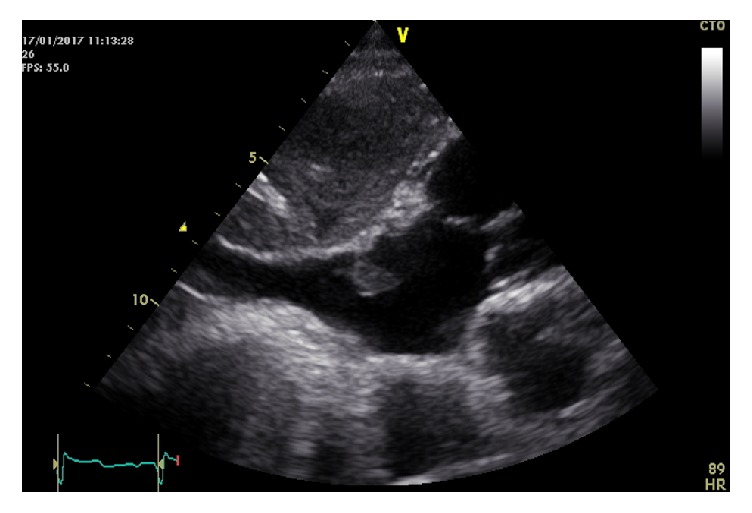
Transthoracic echocardiogram (subcostal window) with mobile vegetation attached to Eustachian valve.

**Figure 2:**
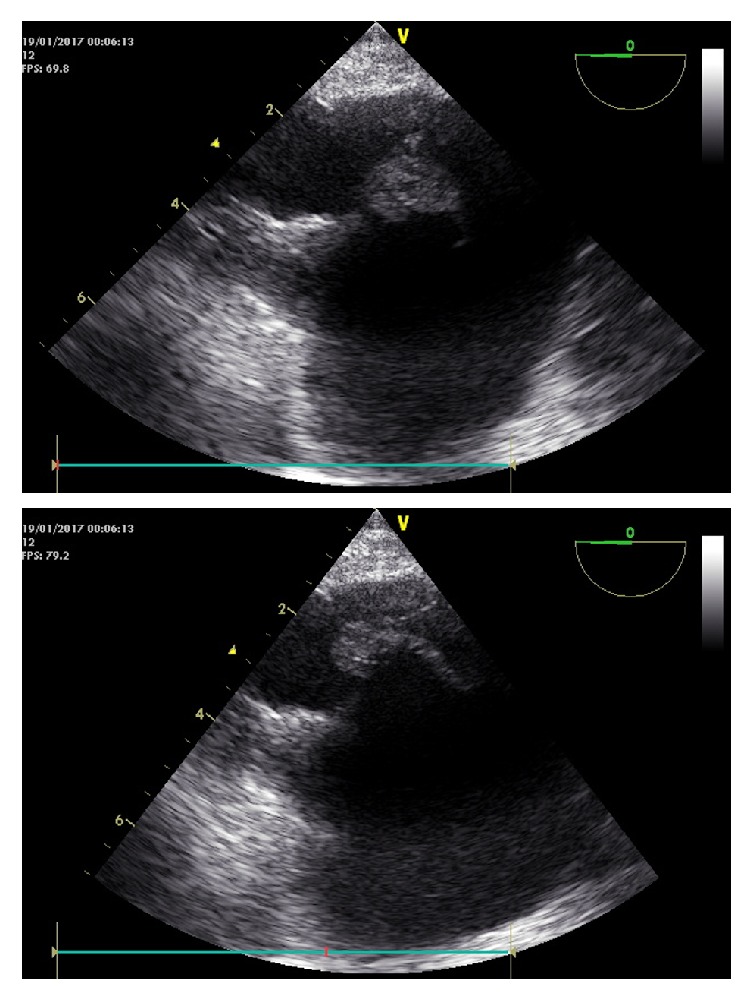
Transesophageal echocardiogram, multilobular heterogeneous vegetation attached to Eustachian valve.

He evolved with combined shock (septic + cardiogenic), needing increasing inotropic support. After 48 hours, a subcostal view of the inferior vena cava joining the right atrium in a second TTE detected a prominent Eustachian valve with mobile multilobulated vegetation attached (Figure 1; Supplementary Materials (available here)). Transesophageal echocardiography at a lower transesophageal view, 0°, confirmed a 12 mm oscillating large mass attached to a visible Eustachian valve (Figure 2); endocarditis was confirmed as the cause of septic shock versus infected Eustachian valve myxoma.

Blood cultures identified methicillin-sensitive* Staphylococcus aureus*.

Repetition of TTE detected a reduction in the size of the vegetation, favoring the diagnosis of Eustachian valve endocarditis.

Even after he was given effective antibiotic treatment (empirical Vancomycin and Gentamicin followed by targeted therapy with Flucloxacillin), the patient deteriorated progressively with refractory shock and died.

## 3. Discussion

Eustachian valve endocarditis (EVE) was firstly described in 1986. From 1986 till 2015, only 29 cases have been reported, and there is only one mention of ICU admission [1].

A recent literature review showed that the main predisposing factor is intravenous drug use and the causative agent is* Staphylococcus aureus*. Other common causes are indwelling catheters, rheumatic heart disease, pacemaker wires, and immunologic compromise [1]. The increasing prevalence of indwelling catheters/devices is hypothesized to be the cause of increasing incidence of Eustachian valve endocarditis in the elderly and expanding spectrum of microbial agents [2].

Due to its location, Eustachian valve is easily seen by transesophageal and transthoracic echocardiography when a subcostal view is used [2, 3]. In our experience the later view has often better resolution in the intensive care unit than in the ward, given the sedation and the positive pressure ventilation. In our case the image was so impressive that it could be seen in both exams. We believe that the first TTE could have neglected this image, since it was accessible only through subcostal window and was not visible in the apical view.

EVE usually follows a benign clinical course, resolving with 4–6-week course of antibiotic [2]. The complexity and severity of our patient with severe aortic stenosis and multiple organ dysfunction dictated his death.

## Supplementary Materials

Supplementary Materials Video 1: transthoracic echocardiogram (subcostal window) with mobile vegetation attached to Eustachian valve.
